# The top hat procedure does not impact the management of women treated by LEEP in cervical cancer screening

**DOI:** 10.61622/rbgo/2024rbgo44

**Published:** 2024-05-27

**Authors:** Juliana Yoko Yoneda, Aline Evangelista Santiago, Julio Cesar Teixeira, Helymar Costa Machado, Sophie Derchain, Milena Yonamine, Diama Bhadra Vale

**Affiliations:** 1 Universidade de Campinas Department of Obstetrics and Gynecology Campinas SP Brazil Department of Obstetrics and Gynecology, Universidade de Campinas, Campinas, SP, Brazil.; 2 Universidade de Santo Amaro Medicine School São Paulo SP Brazil Medicine School, Universidade de Santo Amaro, São Paulo, SP, Brazil.

**Keywords:** Uterine cervical neoplasms, Cervical intraepithelial neoplasia, Colposcopy, Conization, Gynecologic surgical procedures, Cervix uteri, Electrosurgery

## Abstract

**Objective:**

To describe Top-hat results and their association with margin status and disease relapse in a referral facility in Brazil.

**Methods:**

A retrospective study of 440 women submitted to LEEP to treat HSIL, in which 80 cases were complemented immediately by the top hat procedure (Top-hat Group - TH). TH Group was compared to women not submitted to Top-hat (NTH). The sample by convenience included all women that underwent LEEP from January 2017 to July 2020. The main outcome was the histological result. Other variables were margins, age, transformation zone (TZ), depth, and relapse. The analysis used the Chi-square test and logistic regression.

**Results:**

The TH Group was predominantly 40 and older (NTH 23.1% vs. TH 65.0%, p<0.001). No difference was found in having CIN2/CIN3 as the final diagnosis (NTH 17.0% vs. TH 21.3%, p=0.362), or in the prevalence of relapse (NTH 12.0% vs. TH 9.0%, p=0.482). Of the 80 patients submitted to top hat, the histological result was CIN2/CIN3 in eight. A negative top hat result was related to a negative endocervical margin of 83.3%. A CIN2/CIN3 Top-hat result was related to CIN2/CIN3 margin in 62.5% (p=0.009). The chance of obtaining a top hat negative result was 22.4 times higher (2.4-211.0) when the endocervical margin was negative and 14.5 times higher (1.5-140.7) when the ectocervical margin was negative.

**Conclusion:**

The top hat procedure did not alter the final diagnosis of LEEP. No impact on relapse was observed. The procedure should be avoided in women of reproductive age.

## Introduction

Treatment of precursor lesions is a major strategy for cervical cancer prevention. The World Health Organization (WHO) made a global call to eliminate cervical cancer and advocates that 90% of women with positive screening tests should receive treatment.^(1)^ Despite the advances in screening and treatment, cervical cancer remains one of the leading causes of death among women in low- and middle-income countries.^(2,3)^

Loop electrosurgical excision procedure (LEEP) is a widespread treatment of cervical intraepithelial neoplasia (CIN) since its first description in 1989.^(4)^ The main focus of excision is to remove the entire lesion, preventing the development of invasive disease. Meanwhile, it provides the most reliable biopsy specimens and can exclude invasive disease.^(5)^ Incomplete excision and high-risk HPV infection persistence are the most important predictors of recurrence risk.^(5)^

The top-hat procedure is a technique of type 3 excision of the cervix, consisting of a second pass of a deeper resection into the endocervical canal with a small square loop immediately after a traditional LEEP. It aims to reduce incomplete excision and residual disease.^(6,7)^

This study aimed to describe the top-hat procedure's results and association with margin status and disease relapse in a referral facility in Brazil. The results of this study can support the decision on the management of patients in the context of cervical cancer prevention.

## Methods

This is a retrospective study of 440 women submitted to LEEP to treat precursor lesions from January 2017 to July 2020 in the context of cervical cancer screening. In 80 cases, an additional endocervical fragment was obtained after the conventional LEEP procedure (Top-hat Group - TH).

The subjects were women relying on the public health system living in the metropolitan region of Campinas, a heavily populated urban area in São Paulo state, Brazil. They were referred to the University of Campinas Women's Hospital, where medical records were reviewed. The sample was selected by convenience, including all women who underwent LEEP in the period.

Women were referred by cytology and/or biopsies showing high-grade squamous intraepithelial lesion (HSIL), atypical squamous cells cannot exclude high-grade squamous intraepithelial lesion (ASC-H), atypical glandular cells (AGC) or adenocarcinoma *in situ* (AIS) – HSIL+; or by the persistence of atypical squamous cells of undetermined significate (ASC-US) or low grade squamous intraepithelial lesion (LSIL). At admission, women were submitted to colposcopy and referred to LEEP when there was no suspicion of micro-invasive or invasive cancer. LEEP is performed in the outpatient clinic under local anesthesia by colposcopy vision. Top Hat is performed when there is a subjective impression of positive margins in the main fragment, avoiding it in women in their reproductive ages.

In the selected period, 734 LEEPs were performed. The exclusion criteria were cases referred by persistent ASC-US or LSIL (97 cases); micro-invasive or invasive lesions suspected on cytology or colposcopy (28 cases); history of a previous excisional procedure (59 cases); LEEP margins not evaluated due to piece fragmentation (six cases); when the size of the loop used was not recorded (20 cases); and when the final diagnosis in LEEP was micro-invasive or invasive neoplasia (48 cases) or negative (in the main and Top-hat fragment when applied - 36 cases). The final sample consisted of 440 patients.

The final histological diagnosis in the main LEEP fragment, the top-hat fragment, or the endocervical/ectocervical margins was categorized as negative (negative or LSIL/cervical intraepithelial neoplasia grade 1 – CIN 1) or positive (CIN 2 or 3, AIS or more severe). Age was categorized as younger or older than 40 (<40 or ≥40). The transformation zone was classified into types 1, 2, or 3 (TZ 1, 2, or 3), according to IFCPC 2011 colposcopic terminology, and obtained from the procedure form (6). The depth of the main fragment was categorized as 10mm or lower or greater than 10mm (≤10 or >10mm). A re-excision was performed in 48 cases when relapse was suspected by cytology, colposcopy and/or biopsy. In some cases, a re-excision was performed when margins were positive to avoid loss in follow-up in a vulnerable woman. The average time from LEEP to re-excision was 14.9 months, 16.0 months in the Top-hat group, and 14.6 months in the non-top-hat group. Relapse was determined during follow-up by a positive cytology or biopsy/re-excision result showing HSIL+.

For statistical analysis, the description was performed by frequencies using the Chi-square or Fisher's exact test and uni and multivariate logistic regression to estimate Odds Ratio and its 95% Confidence Interval (CI). A significance level of 5% (p<0.05) was adopted. Statistical analyses were performed using SAS 9.4 (SAS Institute Inc. 2013. Cary, NC, USA).

The project was approved by the "Ethics and Research Committee" of the University of Campinas 2.913.889, under the number CAAE 13034013.6.0000.5404 version 3, on September 24, 2018. The Committee waived the need for informed consent.

## Results

The Top-hat procedure was performed in 80 of the 440 cases analyzed (18.8%). When compared to women not submitted to Top-hat (NTH), those who had the complementation were predominantly 40 years and older (NTH 23.1% vs. TH 65.0%, p<0.001), and presented more TZ type 3 (NTH 10.0% vs. TH 37.2%, p<0.001). Having CIN 2 or CIN 3 as the final diagnosis was not significantly different when performing or not the Top-hat (NTH 17.0% vs. TH 21.3%, p=0.362). Indeed, no difference was found in the prevalence of relapse (NTH 12.0% vs. TH 9.0%, p=0.482) (Table 1).

**Table 1:** Description of variables related to LEEP, according to the complementation or not of an immediate second deeper procedure (Top-Hat)

Variables		Top Hat No n(%) 360(100)	Top Hat Yes n(%) 80(100)	Total	p-value
Age					
	<40	277(76.94)	28(35.00)	305	<0.001
	≥40	83(23.06)	52(65.00)	135	
Transformation Zone†					
	Type 1	242(67.79)	27(34.62)	269	<0.001
	Type 2	76(21.29)	22(28.21)	98	
	Type 3	39(10.92)	29(37.18)	68	
Final diagnosis					
	Negative/CIN1	29(8.06)	5(6.25)	34	0.584
	CIN2/CIN3	331(91.94)	75(93.75)	406	
Deepness (main fragment)					
	≤10mm	195(54.17)	39(48.75)	234	0.380
	>10mm	165(45.83)	41(51.25)	206	
Endocervical margin					
	Negative	299(83.06)	63(78.75)	362	
	Positive	61(16.94)	17(21.25)	78	0.362
Ectocervical margin					
	Negative	271(75.28)	63(78.75)	334	0.511
	Positive	89(24.72)	17(21.25)	106	
Re-excision result					
	Negative	13(33.33)	5(55.56)	18	0.529
	HSIL+	24(61.54)	4(44.44)	28	
	SCC	2(5.13)	0(0.00)	2	
Relapse†					
	No	272(88.03)	61(91.04)	333	0.482
	Yes	37(11.97)	6(8.96)	43	

Median time to relapse: Top-Hat No 757.66 months (SD 380.36); Top Hat Yes 742.22 (SD 337.80); p=0.915;

† Missing information in some cases; <40 – women younger than 40; ≥40 – women 40 or older; HSIL+ – cervical intraepithelial neoplasia grade 2 or more severe; SCC – squamous cell carcinoma; SD - standard deviation

Of 80 patients submitted to top hat, in eight (10%), the pathological result of this specimen was CIN 2 or 3. No case had an invasive result in the top hat fragment. Table 2 shows differences observed in cases regarding the Top-hat result. The only significant difference was related to the result of the endocervical margin in the main specimen: a negative top hat result was associated with a negative endocervical margin for cin 2 or cin 3 in 83.3% of cases, and when the top hat result was CIN 2 or CIN 3, it shared the same endocervical margin status in 62.5% of cases (p=0.009).

**Table 2:** Relation of variables and pathological result of the immediate second deeper procedure (Top Hat) in women who have undergone LEEP

Variables		Top Hat result			p-value
		Negative n(%) 72(100)	CIN 2 or CIN 3 n(%) 8(100)	Total n	
Age					
	<40	27(37.50)	1(12.50)	28	0.250
	≥40	45(62.50)	7(87.50)	52	
Transformation Zone†					
	Type 1	24(34.29)	3(37.50)	27	0.203
	Type 2	18(25.71)	4(50.00)	22	
	Type 3	28(40.00)	1(12.50)	29	
Final diagnosis					
	Negative	5(6.94)	0(0.00)	5	1.000
	CIN 2 or CIN 3	67(93.06)	8(100.00)	75	
Deepness (main fragment)					
	≤10mm	33(45.83)	6(75.00)	39	0.150
	>10mm	39(54.17)	2(25.00)	41	
Endocervical margin					
	Negative	60(83.33)	3(37.50)	63	0.009
	Positive	12(16.67)	5(62.50)	17	
Ectocervical margin					
	Negative	59(81.94)	4(50.00)	63	0.058
	Positive	13(18.06)	4(50.00)	17	
Re-Excision result					
	Negative/CIN1	4(66.67)	1(33.33)	5	0.524
	CIN2/CIN3	2(33.33)	2(66.67)	4	
Relapse†					
	No	56(93.33)	5(71.43)	61	0.115
	Yes	4(6.67)	2(28.57)	6	

Median time to relapse: Negative/CIN1 752.43 (SD 327.86); CIN2/CIN3 654.71 (SD 433.84); p=0.448;

† Missing information in some cases; <40 – women younger than 40; ≥40 – women 40 or older; CIN – cervical intraepithelial neoplasia; SD - standard deviation Source: Prepared by the authors.

Looking at the chance of having a Top-Hat negative pathology result, the regression analysis showed that only margin status of the main fragment would predict the result. The multivariate analysis revealed a chance of obtaining a top-hat negative result 22.36 times higher (95% CI 2.37-211.02) when the endocervical margin was negative and 14.50 times higher (95% CI 1.50-140.68) when the ectocervical margin was negative (Table 3).

**Table 3:** Factor related with the chance of having a negative pathology result of the immediate second deeper procedure (Top Hat) in 72 women who have undergone LEEP

Variables		Univariate analysis			Multivariate analysis		
		p-value	OR	95% CI	p-value	OR	95% CI
Age							
	<40	—	1.00	—			
	≥40	0.191	0.24	0.03 – 2.04			
Transformation Zone							
	Type 1	—	1.00	—			
	Type 2	0.486	0.56	0.11 – 2.83			
	Type 3	0.292	3.50	0.34 – 35.90			
Final Diagnosis							
	Negative	—	1.00	—			
	CIN 2 or CIN 3	0.444	1.39	0.07 – 27.32			
Deepness (Main Fragment)							
	≤10mm	—	1.00	—			
	>10mm	0.137	3.55	0.67 – 18.76			
Endocervical Margin							
	Positive	—	1.00	—	—	1.00	—
	Negative	0.008	8.33	1.75 – 39.65	0.007	22.36	2.37 – 211.02
Ectocervical Margin							
	Positive	—	1.00	—	—	1.00	—
	Negative	0.049	4.54	1.01 – 20.55	0.021	14.50	1.50 – 140.68

Analysis by Logistic Regression with Stepwise criteria; p-value; <40 – women younger than 40; ≥40 – women 40 or older; CIN – cervical intraepithelial neoplasia; SD: standard deviation

## Discussion

In this retrospective study of 440 women submitted to LEEP for HSIL treatment, the top hat procedure was performed in 80. Top-hat cases were mainly women older than 40 and, as expected, presented more TZ type 3. CIN 2 or CIN 3 as the final or more severe diagnosis was not significantly different when performing Top-hat or not. The procedure showed no impact on relapse.

The role of the additional procedure after LEEP by Top-hat or endocervical sampling is still unclear. Cejtin et al.^(8)^ found that additional procedures for endocervix sampling had no prognostic insight. At the same time, Cui et al.^(9)^ concluded that endocervical sampling had a higher predictive value than margins to predict residual or persistent disease. Chen et al.^(10)^ found that a positive top hat specimen was associated with short-term treatment failure.

Of the 80 cases submitted to the top hat procedure, only eight (10%) showed CIN2 or CIN3 on the pathological analysis. A negative top hat result was associated with a negative endocervical margin in the main fragment in 83.3% of cases. When the Top-hat result was CIN 2 or CIN 3, the endocervical margin in the main fragment was positive in 62.5% of cases. The chance of having a negative Top-hat result was 22 times higher when the endocervical margin was negative and 15 times higher when the ectocervical margin was negative.

The role of endocervical margin in predicting relapse is well reported.^(11–14)^ A balance between oncologic safety and obstetrics morbidities can be expected when relating the type of excision with the TZ type.^(6,15–18)^ In three cases submitted to the top hat these specimens showed residual HSIL+, and the endocervical margin of the main fragment was negative. However, the role of residual disease is controversial in predicting relapse. The healing provocated by the scar is probably the mechanism for justifying the dissipation of the residual lesion during follow-up. Important to note that residual disease or relapse should be a weak argument for deeper incisions in young women because, even when margins are positive, the relapse rate is low.^(5,12–14)^

Top hats were performed on 28 of 305 women younger than 40 (8%). In fact, 17% of women with negative endocervical margins were submitted to top hat. Considering the lack of evidence to support the benefits of the top hat procedures, those women were mistakenly submitted to the potential risks of adverse obstetrics outcomes. The literature shows that women with CIN have a higher risk for prematurity and that excisional treatment can increase that risk, directly related to the deepness of the excisions.^(19)^ Top hat should not be recommended to women in their reproductive ages.

Older women are at a higher risk for recurrence^(5,18)^ and may have some benefit in two situations: when the glandular disease is suspected or when TZ is type 3. In both situations, the top hat should be evaluated if the first loop is lower than 15mm (excision types 1 or 2), or when the first pass could not technically reach 10-15mm of deepness. The top hat procedure in this situation may prevent excessive bleeding or infection risk due to a lower stroma removal when compared to a loop that is 20 or 25mm high.

The strength of this study is that it reinforces the value of clinical findings and provides quality information for colposcopists to improve the treatment of cervical intraepithelial neoplasia. The main limitation is its retrospective nature - we did not perform the Top-hat procedure randomly or follow subjective criteria for its election. There was a potential selection bias since the Top-hat procedure has been performed on older women at our institution. Another limitation is the lack of HPV testing information, which is prevailing in most low- and middle-income countries. Further prospective studies could analyze the prognostic value of the Top-hat procedure. However, considering the obstetrics risks, there is a lack of support in the literature if the design includes young women.

## Conclusion

In this retrospective evaluation of the top hat procedure, we observed that a second pass after LEEP did not alter the final diagnosis. No impact on relapse was observed. The procedure should be avoided in women in their reproductive ages. Considering the obstetrics morbidities and the value of the HPV status on follow-up, we believe there is no support to design a prospective study.
